# Maize Transformation: From Plant Material to the Release of Genetically Modified and Edited Varieties

**DOI:** 10.3389/fpls.2021.766702

**Published:** 2021-10-14

**Authors:** Juliana Erika de Carvalho Teixeira Yassitepe, Viviane Cristina Heinzen da Silva, José Hernandes-Lopes, Ricardo Augusto Dante, Isabel Rodrigues Gerhardt, Fernanda Rausch Fernandes, Priscila Alves da Silva, Leticia Rios Vieira, Vanessa Bonatti, Paulo Arruda

**Affiliations:** ^1^Embrapa Informática Agropecuária, Campinas, Brazil; ^2^Genomics for Climate Change Research Center (GCCRC), Universidade Estadual de Campinas, Campinas, Brazil; ^3^Centro de Biologia Molecular e Engenharia Genética, Universidade Estadual de Campinas, Campinas, Brazil; ^4^Departamento de Genética, Evolução, Microbiologia e Imunologia, Instituto de Biologia, Universidade Estadual de Campinas, Campinas, Brazil

**Keywords:** maize, plant transformation, gene editing, plant biotechnology, genetic modification, morphogenic regulator-mediated transformation

## Abstract

Over the past decades, advances in plant biotechnology have allowed the development of genetically modified maize varieties that have significantly impacted agricultural management and improved the grain yield worldwide. To date, genetically modified varieties represent 30% of the world’s maize cultivated area and incorporate traits such as herbicide, insect and disease resistance, abiotic stress tolerance, high yield, and improved nutritional quality. Maize transformation, which is a prerequisite for genetically modified maize development, is no longer a major bottleneck. Protocols using morphogenic regulators have evolved significantly towards increasing transformation frequency and genotype independence. Emerging technologies using either stable or transient expression and tissue culture-independent methods, such as direct genome editing using RNA-guided endonuclease system as an *in vivo* desired-target mutator, simultaneous double haploid production and editing/haploid-inducer-mediated genome editing, and pollen transformation, are expected to lead significant progress in maize biotechnology. This review summarises the significant advances in maize transformation protocols, technologies, and applications and discusses the current status, including a pipeline for trait development and regulatory issues related to current and future genetically modified and genetically edited maize varieties.

## Introduction

As an important global crop and a model plant for genetics and biotechnology studies, maize is one of the most researched plant species. Because of its richness in genetic and genomic resources, maize has been used for biological investigations into plant domestication and evolution, epigenetics, heterosis, disease, insect resistance inheritance, doubled haploids, genome editing, and breeding tools (Strable and Scanlon, 2009; Andorf et al., 2019). However, the development of genetically modified maize varieties has faced enormous difficulties due to genotype-associated recalcitrance to transformation. The first protocols for stable maize transformation, which were published in the late 1980s, used particle bombardment, but the transformation frequency obtained with the method was very low (Gordon-Kamm et al., 1990; Frame et al., 1994). Nevertheless, agricultural biotechnology companies were able to launch commercial transgenic varieties using this protocol (ISAAA database, 2021). A few years later, the overwhelming success of herbicide- and insect-resistant transgenic maize varieties modified the global seed industry from a pulverized market with several local and small seed companies into a consolidated market with a few transnational companies able to invest in research and bear the expensive regulatory costs to commercialize genetically modified varieties (Bijman, 2001; Howard, 2009). In 2019, genetically modified maize varieties accounted for over 30% of the world’s maize cultivated area (ISAAA database, 2021). The list of the so-called biotech traits currently available is no longer restricted to herbicide and insect resistance; abiotic stress tolerance, high yield, and improved nutritional quality are traits expected to be introduced into the market soon (Wu et al., 2019; Simmons et al., 2020; ISAAA database, 2021).

The progress in maize transformation protocols over the years aimed to overcome genotype recalcitrance and produce less complex and unfragmented transgene insertions in the plant genome, which is a major drawback associated with the regulatory aspects of transgenics (Kausch et al., 2021a). As a result, *Agrobacterium*-mediated transformation has become the most suitable protocol for selecting single and unfragmented insertion events. Increasing the transformation frequency and expanding the number of genotypes suitable for *Agrobacterium* infection and plant regeneration of transgenic events were the focus of research groups in both the industrial and academic sectors (Frame et al., 2002, 2006; Ishida et al., 2003, 2007; Huang and Wei, 2005; Lowe et al., 2018). In parallel to improvements in protocols for *Agrobacterium* infection and transformation, advances have also been achieved in plasmid design, suitable promoters, and selectable marker genes (Kausch et al., 2021b; Simmons et al., 2021). One of the most significant advances that resulted in improvements in maize transformation was the design of constructs expressing morphogenetic regulators (MRs) that allow direct embryogenesis from immature zygotic embryos (IZEs) and thereby bypass the callus induction stage (Lowe et al., 2016, 2018; Svitashev et al., 2016; Mookkan et al., 2017; Barone et al., 2020). For example, vector constructs expressing the MRs BABY BOOM and WUSCHEL allowed the transformation of elite maize inbred lines at frequencies of up to 50%, and this process bypasses the laborious and time-consuming backcross programmes for introgression of the transgene into commercial hybrids (Lowe et al., 2016).

The costs associated with the deregulation of genetically modified commercial maize plants are prohibitive for most public research institutions. Only the largest agricultural biotechnology companies are financially prepared to pay these costs, and therefore, the world has seen an increasing concentration of maize seed providers (Deconinck, 2019). This scenario may be overcome by gene-editing technologies that simplify gene structure/expression manipulation (Jorasch, 2020). Provided that regulatory agencies worldwide become aware of the potential of extensively using this technology, gene editing will soon become more accessible to the public interested in contributing to agricultural sustainability, which will allow the worldwide development of biotechnology varieties that incorporate desirable traits (Schiemann et al., 2019). Gene-editing technologies will also benefit from the progress being made in DNA and protein delivery mechanisms and tissue culture-free methods for maize modification (Li et al., 2017; Vejlupkova et al., 2020). For example, double haploid induction associated with gene editing methods [simultaneous double haploid production and editing (Hi-Edit) and haploid-inducer mediated genome editing (IMGE)] opens new opportunities to speed up precision breeding (Kelliher et al., 2019; Wang et al., 2019). The possibility of obtaining genetically modified plants without integrating foreign DNA also opens a new path for the deregulation of biotech traits. In several countries, including Argentina, Brazil, and the USA, genome-edited varieties that do not incorporate foreign DNA have already been deregulated as conventional improved varieties with no additional restrictions (Hull et al., 2021). The potential lack of a need for approval by regulatory agencies would significantly reduce the time and costs to introducing the new edited varieties to the market compared with those needed for regulated transgenic varieties (Lassoued et al., 2019).

Recent advances in the transformation and regulatory aspects of genetically modified/edited maize plants have encouraged academic and private facilities to provide transformation services, which has allowed small research groups and companies to test their genes and alleles. In addition, the genome sequences of several maize lines and hybrids are already available (Hufford et al., 2021), which allows the design of strategies for genetic modification/edition using improved transformation protocols. These improvements will allow the evaluation of an increasing number of genes and alleles associated with desirable agronomic traits (Portwood et al., 2019).

As a critical technology, maize transformation has been the central theme of several reviews covering different aspects ranging from historical and current advances in transformation protocols, methods, and applications (Wang et al., 2009; Que et al., 2014; Yadava et al., 2017; Ishida et al., 2020; Kausch et al., 2021a, 2021b). Here, we present the latest advances in the protocols and technologies for maize transformation and expand the topic to the development of new genetically engineered maize varieties, regulatory issues, and the importance of delivering new commercial biotech maize varieties to the market.

## Current Status of Maize Transformation

### Plant Genotype

One of the bottlenecks associated with *Agrobacterium*-mediated transformation is the recalcitrance of maize to bacterial infection and the regeneration of transformed plants. Almost all published protocols have yielded the successful transformation of a few genotypes that usually exhibit satisfactory *Agrobacterium* infection, callus formation, and plant regeneration (Ishida et al., 1996, 2003; Frame et al., 2006; Coussens et al., 2012). The most commonly used maize genotype in academic laboratories is the single hybrid Hi-II (Armstrong et al., 1991) and their inbred parents (Frame et al., 2006; Ishida et al., 2007). However, although these genotypes show high-frequency *Agrobacterium* infection of IZEs, embryogenic callus formation, good performance in selective medium, and recovery of transformed plants, they lack the minimal agronomic performance needed for phenotyping characterization (Frame et al., 2006; Wang et al., 2009; Kausch et al., 2021a). In addition, events generated from individual embryos produced by self-pollinated Hi-II hybrid plants have different genetic backgrounds. These constraints require time-consuming backcross programmes for the introgression of transgenic events into elite inbred lines for phenotypic evaluation. More recently, the B104 maize inbred line has been used for maize transformation. Although this genotype presents a slightly lower transformation frequency, the plants have better agronomic performance, which allows phenotyping of the transformed plants at the T_1_ generation. In general, the transformed B104 plants are vigorous and produce a high number of kernels in the T_1_ generation, which allows the selection of homozygous transformed alleles when self-pollinated (Frame et al., 2006; Coussens et al., 2012; Raji et al., 2018). The B104 maize inbred line, when crossed with elite lines, gives rise to single hybrids that show suitable yield performance in field trials (Feys et al., 2018). Companies usually utilize their proprietary elite genotypes, such as NP2222 (Zhong et al., 2018), PHR03, PH184C, and PH1V69 (Simmons et al., 2021). Tropical maize genotypes have also been transformed (Yadava et al., 2017). In general, the transformation efficiency was lower than that of the temperate genotypes with the exception of that reported for the Sudanese inbred line IL3, which reached 3.78% compared with 0.98% of the A188 inbred line (Omer et al., 2013).

### Explant Material

Genetic transformation requires the efficient introduction of a DNA construct harbouring target and marker genes into the plant cell, which is an effective tissue culture and plant regeneration protocol that allows selection of the transformed cell/tissue, and the further development of fertile plants. The first maize transformation protocols used cell suspensions and calli as explants, and the DNA construct was delivered by particle bombardment (Gordon-Kamm et al., 1990). A few years later, successful maize transformation was achieved using *Agrobacterium tumefaciens* carrying a modified bacteria Ti plasmid harbouring a gene conferring antibiotic resistance (Ishida et al., 1996). Nevertheless, the transformation frequency achieved with either particle bombardment or *Agrobacterium* was very low. In the following years, the construct design was improved to incorporate high-expression promoters to drive selectable marker and target gene expression, which, along with improvements in the culture media and infection treatments, increased the proportion of single-copy insertion events and made *Agrobacterium* the best choice for maize transformation (Frame et al., 2002; Kausch et al., 2021b). In general, the improved protocols made use of the highly efficient *Agrobacterium* strains EHA101 (Hood et al., 1986), EHA105 (Du et al., 2019), and LBA4404 (Ishida et al., 1996, 2007; Zhong et al., 2018). Although attempts have been made to use different explants (Mu et al., 2012), almost all current protocols use IZEs for *Agrobacterium*-mediated transformation due to the well-established callus induction and somatic embryogenesis obtained with this explant (Wang et al., 2009; Yadava et al., 2017; Kausch et al., 2021b). IZEs give rise to type II callus-induced somatic embryos that, upon efficient selection, produce regenerated transformed events (Kausch et al., 2021a).

Although IZEs are the best explant choice for maize transformation, care should be taken regarding aspects that affect transformation frequency. First, maize plants must be grown in a greenhouse with environmental control to ensure the homogeneous growth of healthy and vigorous plants, particularly for routine transformation throughout the year (Ishida et al., 2020). Detailed protocols for growing maize in greenhouses are available in the literature (Eddy and Hahn, 2012), but adjustments to the temperature, light quality/intensity, optimized nutritional conditions, and disease controls are often needed to ensure the quality of IZE production. Second, for classical *Agrobacterium*-mediated transformation, fresh embryos varying in size between 1.2mm and 2mm should be used (Raji et al., 2018). It is important to note that the embryo size usually varies within a single ear, and very small or larger embryos thus need to be discarded.

### Culture Media

In general, maize transformation protocols use culture media prepared with N6 or MS salts (Frame et al., 2006). The protocols can be optimized by altering the combinations of sugars, salts, vitamins, amino acids, antioxidants, antibiotics, and growth regulators (Ishida et al., 2003; Frame et al., 2006; Yadava et al., 2017). Supplementation with silver nitrate has resulted in increased embryogenic callus induction and the recovery of regenerated plants (Ishida et al., 2003; Wang et al., 2009). The combination of copper sulfate (CuSO_4_) with 6-benzyl amino purine (BAP) has been shown to increase embryogenic callus induction and plant regeneration (Cho et al., 2014), whereas the combination of BAP with cysteine and dithiothreitol increases the infection rate (Du et al., 2010). Factors influencing *Agrobacterium*-mediated transformation include the concentration of the virulence inducer acetosyringone, the cocultivation time, and preinoculum bacterial growth (Du et al., 2019).

The effective concentrations of selective agents (antibiotics, herbicides, and sugars) should be optimized to inhibit the growth of nontransformed cell clusters (Wang et al., 2009; Que et al., 2014; Dong et al., 2021). In addition, nontransformed callus sectors that commonly grow around the transformed cell clusters should be removed to increase the recovery of transformed, regenerated plants (Raji et al., 2018). The selectable marker genes used in the constructs designed for maize transformation include antibiotic-resistant *neomycin phosphotransferase II* (*nptII*; Breyer et al., 2014; Barone et al., 2020; Hoerster et al., 2020), hypoxanthine phosphoribosyl transferase (*hpt*; Ishida et al., 2007) and the herbicide resistance genes *phosphinothricin N-acetyltransferase* (*pat*/*bar*; Frame et al., 2002; Ishida et al., 2003) and *acetolactate synthase* (*Hra*/*als*; Zhang et al., 2005; Hoerster et al., 2020). Selectable herbicide markers commonly confer a trait that is highly desired in agronomic performance (Que et al., 2014). The use of *bar*/*pat* genes as a selective marker allows the selection of transformed calli with phosphinothricin (PPT) or its derivatives and has been shown to be very effective for the selection of transformed maize plants (Wang et al., 2009; Que et al., 2014; Yadava et al., 2017). Other selection systems based on the metabolism of sugars (mannose) and amino acids (D-serine and D-alanine) have emerged (Que et al., 2014; Yadava et al., 2017).

### Improvement of *Agrobacterium* Strains

*Agrobacterium* strains have been improved to achieve increased plant transformation frequency through the development of binary vectors (Zambryski et al., 1982; Hoekema et al., 1983; Bevan, 1984; Komari et al., 2006), the development of ternary helper plasmids and superbinary vectors (Ishida et al., 1996; Anand et al., 2018; Zhang et al., 2019), the upregulation of virulence (*vir*) gene expression (Ishida et al., 1996; Van Der Fits et al., 2000; Ye et al., 2011; Vaghchhipawala et al., 2018), and the removal of negative factors of T-DNA transfer (Nonaka et al., 2019). Genome editing has also helped improve *Agrobacterium* strains themselves. For example, clustered regularly interspaced short palindromic repeats (CRISPR)-mediated loss-of-function mutations in *rec*A have generated an EHA105 strain with improved performance for maize transformation (Rodrigues et al., 2021). RecA-deficient strains are typically used to avoid the recombination of additional virulence genes from ternary helper plasmids with homologous sequences from the Ti plasmid (Mookkan et al., 2017; Anand et al., 2018; Sardesai et al., 2018), which allows their concomitant use with ternary vectors harbouring additional virulence genes and morphogenic regulators to increase plant regeneration.

Other features may also be manipulated to improve *Agrobacterium*-mediated transformation and the fate of transformed events. For example, the subversion of host plant factors (Pitzschke, 2013; Sardesai et al., 2013; Hwang et al., 2015), the reduction of vector backbone and transposon integration (Ülker et al., 2008; Kim and An, 2012; Jupe et al., 2019), the increase of T-DNA transfer capacity (Nonaka et al., 2019), the environmental containment of *Agrobacterium* when used for field applications (Torti et al., 2021), the increase on transient transformation frequency (Wang et al., 2018) and the use of effective tools for non-invasive monitoring of gene expression and plant transformation (He et al., 2020; Xu et al., 2021).

Another promising perspective is the use of autonomously replicating virus-based vectors (Zaidi and Mansoor, 2017) for overexpression, gene silencing, or gene editing in maize *via Agrobacterium* (Ding et al., 2006; Wang et al., 2016; Jarugula et al., 2018; Gao et al., 2019; Hu et al., 2019; Mei et al., 2019; Xie et al., 2021). These technologies have allowed transient expression at an industrial scale by spraying *Agrobacterium* carrying the viral vector (Torti et al., 2021). Bypassing difficulties, such as low transient gene transfer rates, regeneration difficulties, and host cell integrity issues (Zaidi and Mansoor, 2017; Nonaka et al., 2019), the virus-based vectors could be optimized to allow the development of transient expression strategies for CRISPR-Cas gene editing without the stable integration of foreign DNA.

### Standard Protocol for Maize Transformation

**Figure 1:**
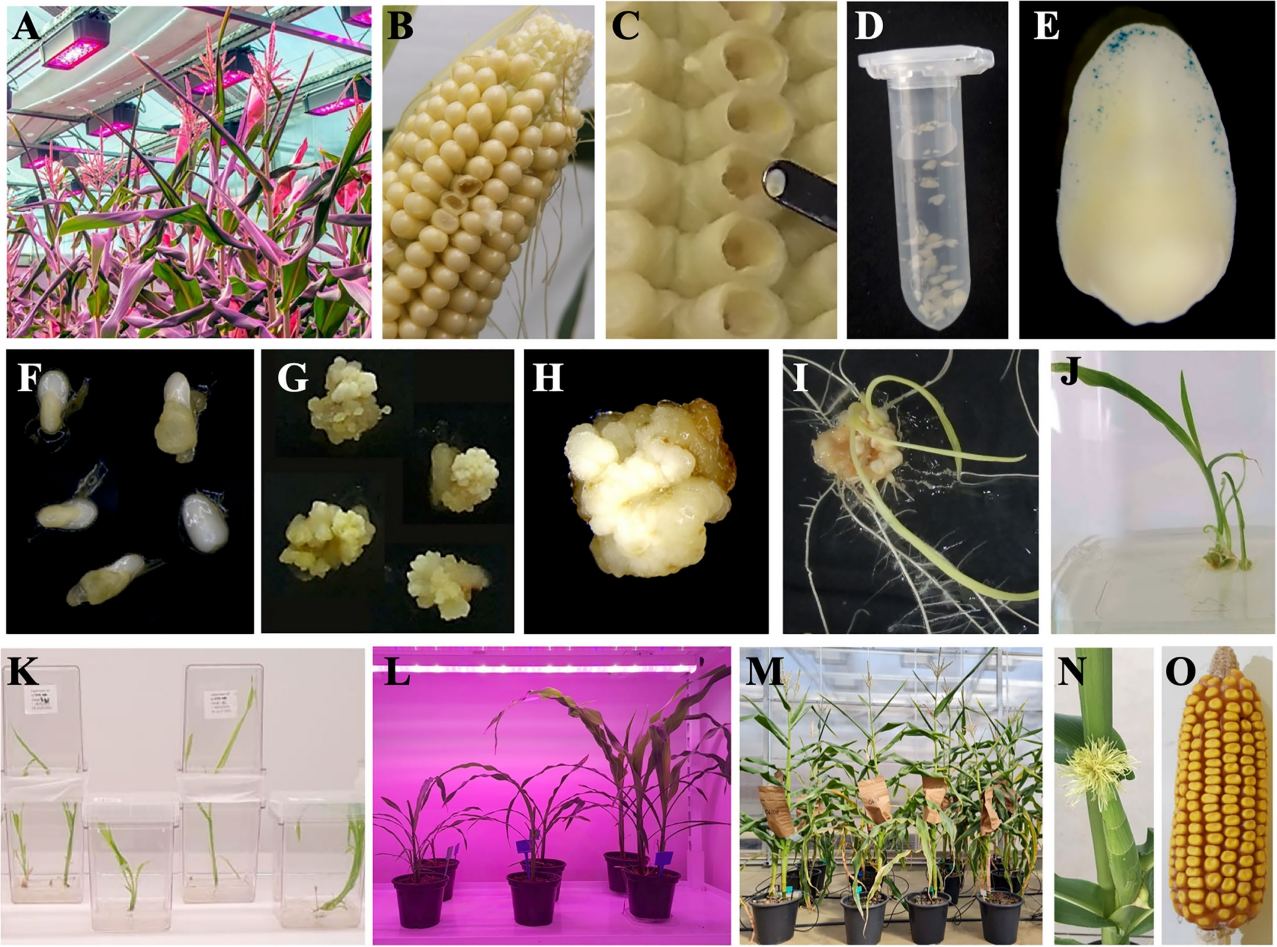
Standard protocol for B104 maize transformation. **(A)** Growth of donor plants for immature embryo production under greenhouse-controlled conditions. **(B)** Ears are harvested 10–16 d after pollination. **(C)** The immature zygotic embryo reaches the ideal size of 1.2–2mm. **(D)** Isolated immature embryos in co-cultivation. **(E)** Immature embryo transiently expressing the *gus* reporter gene. **(F)** Embryos in resting media seven days after *Agrobacterium* infection. **(G)** Calli induction on selection I medium. **(H)** Compact type I callus in selection II medium. (**I,J)** Regeneration of transformed plants. (**K)** Transformed plantlets with roots and shoots are grown in the penumbra room. (**L)** T_0_ transgenic individuals rooted in the soil in an acclimation room. **(M)** T_0_ plants grown at the greenhouse. **(N)** Flowering and pollination of T_0_ plants at the greenhouse. **(O)** Harvesting of T_1_ seeds. The complete process, from infection to T_1_ seed production, takes approximately 6–8months. The images are not to scale.

A basic protocol based on published data (Ishida et al., 1996; Frame et al., 2002; Zhao et al., 2002; Huang and Wei, 2005; Frame et al., 2006; Ishida et al., 2007; Vega et al., 2008; Lee and Zhang, 2016) is currently being used for routine maize transformation using IZEs as explant in many laboratories worldwide. The main steps of this routine maize transformation (Figure 1) involve plant growth under controlled conditions (Figure 1A), harvesting ears at 10–16days post-pollination, selecting 1.2–2-mm IZEs (Figures 1B–C), infecting with *Agrobacterium* harbouring the desired construct (Figure 1D), monitoring the infection rate using GUS-harbouring vectors (Figure 1E), incubating the infected embryos in the dark at 21°C for resting (Figure 1F), transferring the infected embryos to the first-round selective medium in the dark at 25°C (Figure 1G), transferring the infected embryos to the second-round selective medium in the dark at 25°C (Figure 1H), transferring the resistant embryogenic calli to the first-round regeneration medium in the dark at 25°C (Figure 1I), transferring the regenerating plants to second-round regeneration medium to rooting in penumbra light (16hs) at 25°C (Figures 1J–K), transferring the regenerated plants to the acclimation room at 26°C/22°C day/night, 16h light (Figure 1L) and finally transferring the acclimated plants to the greenhouse for plant growth and T_1_ seed production (Figures 1M–O).

When growing plants for IZE harvesting, environmental factors should be taken into account. For the B104 genotype, the recommended greenhouse temperature varies between 20°C and 28°C (night/day), and light intensity varies between 600 and 1,000μmol.m^−2^.s^−1^. Fine-tuning the temperature and light are essential to the production of high-quality embryos for successful transformation. For routine transformation throughout the year, the plants are pollinated weekly, and the IZEs reach their ideal size 10–16days after pollination. The harvested ears can be stored at 4°C for a couple of days prior to use. The *Agrobacterium* strains EHA101, EHA105 and LBA4404 are commonly used for the transformation of IZEs. After infection in N6 liquid medium (Figure 1D), the IZEs are laid down on cocultivation medium for 3days in the dark at 21°C (Figure 1F). The cocultivation medium is similar to the infection medium with the exception that the N6 salts are substituted by MS salts (Frame et al., 2006). The infection occurs mainly in the scutellum cells facing upward (Figure 1E). After 3days in the cocultivation medium, the IZEs are transferred to a resting medium containing the same composition of cocultivation medium with the appropriate antibiotics for bacterial counterselection for 7days at 21°C in the dark (Figure 1F). The IZEs are then transferred to a selective medium containing a low concentration of the PPT selective agent for 14days (Figure 1G) and then to a selective medium containing an increased concentration of the selective agent for 28days (Figure 1H). The selective agent depends on the construct selectable marker being the most popular *bar* gene that confers bialaphos/phosphinothricin (PPT) resistance (Gordon-Kamm et al., 1990; Frame et al., 2002). In this case, PPT is first used at 1,5mg/l for 14days, followed by 28days at 5mg/l. Other selectable marker genes widely used include *nptII*, which confers kanamycin resistance (Breyer et al., 2014; Barone et al., 2020; Hoerster et al., 2020), and *hra*, which confers imazapyr resistance (Hoerster et al., 2020) and *pmi* for resistance to mannose (Dong et al., 2021). In general, embryogenic callus induction is completed after 2weeks on low-concentration selective medium (Figure 1G). Maize genotypes able to produce type II calli exhibit early embryogenesis with the rapid proliferation of somatic embryos, whereas genotypes that produce type I callus genotypes exhibit late embryogenesis with organogenic and meristematic domes showing more compact tissues (Kausch et al., 2021b). After the selection phase, the selected calli are fragmented and transferred to regeneration medium without hormones containing the maximum concentration of selective agent (6mg/l of PPT, Figure 1I) and without hormones for 3weeks and then to regeneration medium without hormones and selective agents at low light intensity for 2–4weeks (Figures 1J–K). Regenerated plantlets with roots and shoots are finally transferred to soil and acclimated until the plants are in appropriate condition to be transferred to the greenhouse (Figures 1L–M). The transformation time, from *Agrobacterium* infection to T_1_ seeds takes approximately 6–8months (Figure 1O).

### Maize Transformation Service Providers

Despite recent advances in maize transformation, only a few academic groups worldwide have the necessary infrastructure to effectively implement maize transformation. Although improved protocols are available, the steps from immature embryos to T_1_ seeds require well-equipped laboratory and greenhouse facilities for the *Agrobacterium*-mediated transformation (Wang et al., 2009; Altpeter et al., 2016). In addition to a high-quality infrastructure, the availability of skilled personnel is another factor determining the success of maize transformation. Due to these constraints, several specialized public and private facilities currently offer maize transformation services (Table 1). B104 and Hi-II are the most used genotypes by maize transformation service providers. Public and private maize transformation providers are available in the USA, Brazil, India, and Europe (Table 1).

**Table 1:** Academic laboratories and facilities providing maize transformation services.

Country	Laboratory/Facility	Maize Genotype	Website
Argentina	INDEAR	NA	https://www.indear.com/en/plant-transformation-and-tissue-culture/
Belgium	VIB Crop Genome Engineering Facility	B104	https://www.psb.ugent.be/cores/crop_genome_engineering_facility
Brazil	Pangeia Biotech	Hi-II	https://www.pangeiabiotech.com/
Germany	Crop Genetic Systems	A188 B104 Hi-II	https://www.crop-genetic-systems.de/english/prices/
India	Metahelix	Tropical/ Temperate	https://www.rallis.co.in/Seed_Division/main/pt_transformation.html
USA	Wisconsin Crop Innovation Center	Hi-II LH244	https://cropinnovation.cals.wisc.edu/services/maize-transformation/
USA	Cornell CALS - College of Agriculture and Life Sciences	B104	https://cals.cornell.edu/school-integrative-plant-science/about/campuses-facilities/plant-transformation-facility
USA	Plant Transformation Core Facility at the University of Missouri	Hi-II B104	https://research.missouri.edu/ptc/services.php
USA	Danforth Center Core Facilities	NA	https://www.danforthcenter.org/our-work/core-facilities/plant-transformation/
USA	Plant Transformation at The University of Rhode Island	Hi-II	https://web.uri.edu/pbl/plant-transformation/
USA	Creative Biogene	Hi-II B104 Others	https://www.creative-biogene.com/services/maize-transformation-service.html

## Emerging Technologies for Maize Transformation

### Overcoming Genotype Recalcitrance: Morphogenic Regulator-Mediated Transformation

**Figure 2:**
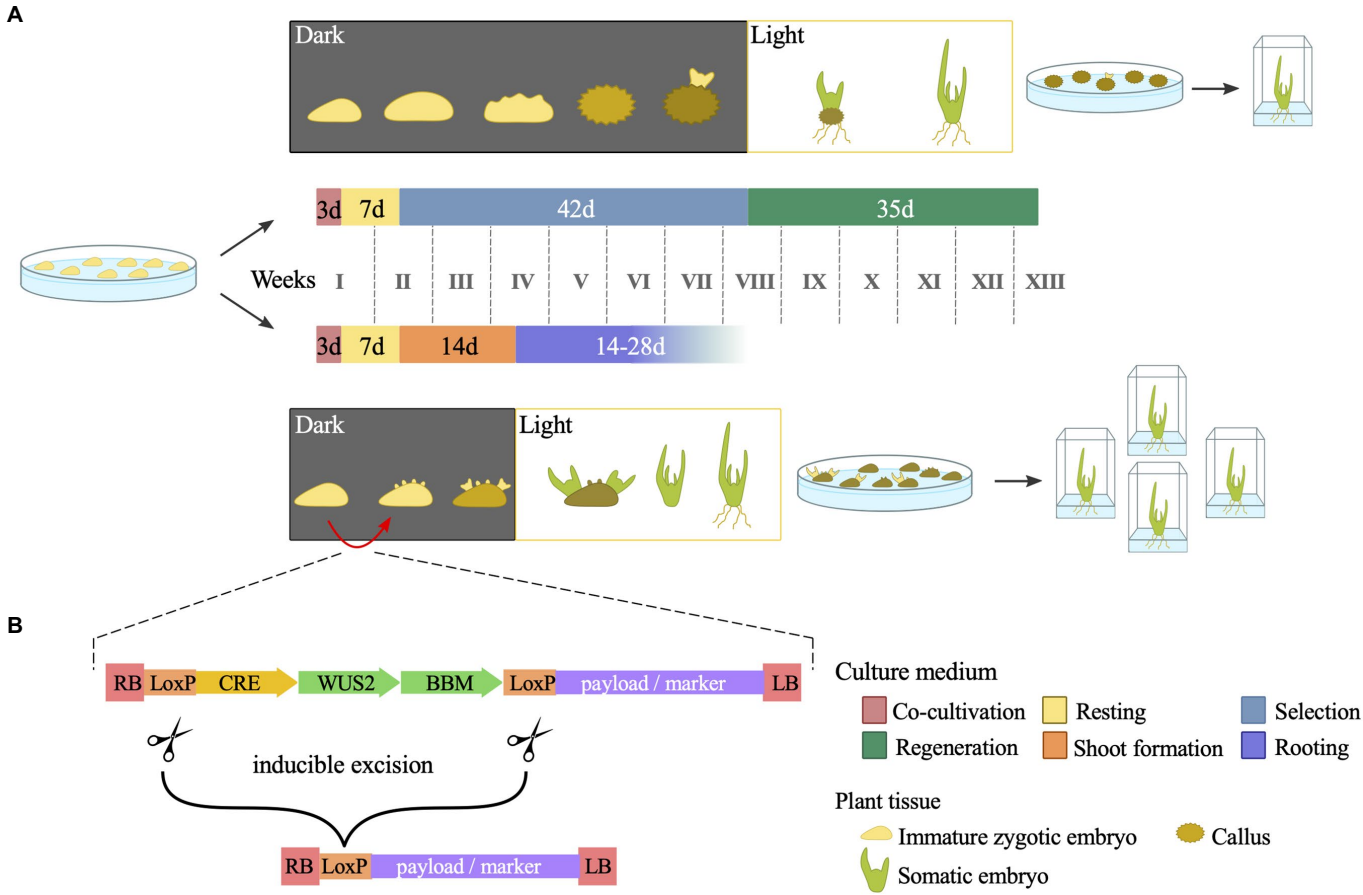
Schematic representation of the morphogenic regulator-mediated maize transformation (MRMT). **(A)** Comparison between standard transformation (top) and MRMT (bottom) protocols. Tissue culture phases are indicated by different colours. By skipping the callus culture step, MRMT shortens the time needed for *in vitro* tissue culture. Note that although not specified, a selective agent is used in the MRMT culture media to allow regeneration of transformed embryos only. **(B)** Schematic representation of generic T-DNA present in MRMT-based vectors. In addition to the genetic payload of interest, T-DNA harbours morphogenic regulators (MRs) and a recombinase (CRE). Upon a given stimulus, CRE excises the MRs from the construct. The time period and culture media are based in Coussens et al. (2012) and Raji et al. (2018) for the standard protocol and in Masters et al. (2020) for the MRMT protocol.

Although routinely performed, maize transformation faces the constraints of a few genotypes amenable to *Agrobacterium*-mediated transformation. The maize inbred line B73, for instance, is an important genetic and genomic resource but is strongly recalcitrant to transformation. The same is true for most of the commercial elite maize inbred lines. In addition, even the most improved transformation protocol currently available requires a callus culture step, which is laborious, time-consuming and constitutes a constraint for efficient large-scale transformation pipelines (Lowe et al., 2016). New methods relying on morphogenic regulators (MRs) expression at the early steps of maize transformation have been developed to overcome these obstacles. MRs, such as *BABY BOOM* (*BBM*), *OVULE DEVELOPMENT PROTEIN 2* (*ODP2*), and *WUSCHEL 2* (*WUS2*), are transcription factors capable of inducing somatic embryogenesis in different plant tissues. Transformation vectors harbouring combinations of *WUS2* with either *BBM* (Lowe et al., 2016, 2018; Mookkan et al., 2017; Barone et al., 2020) or *ODP2* (Svitashev et al., 2016) along with selectable markers and target genes have demonstrated high transformation frequency. In general, these Morphogenic Regulator-Mediated Transformation (MRMT) vectors can be introduced in the current *Agrobacterium* strains and used for immature embryo transformation. The MR methods exhibit two significant benefits: (1) increased plant regeneration rates and the recovery of transformed plants from recalcitrant genotypes and (2) a shortening in the overall time needed for transformation by bypassing the callus culture step (Figure 2A).

The first published MRMT method demonstrated that the use of vectors harbouring *BBM* and *WUS2* for either *Agrobacterium-* or particle bombardment -mediated transformation increased the transgenic events recovery from recalcitrant genotypes by 25 to 52% (Lowe et al., 2016). Soon after, a transformation frequency of 15% was obtained for the recalcitrant B73 inbred line (Mookkan et al., 2017). Despite this transformation success, the continuous expression of MRs can lead to various developmental defects. Thus, the expression of MRs needs to be restricted to the embryogenesis induction step. Two approaches have been developed to overcome these detrimental effects: (1) excision of the MR expression cassette by a recombination system and (2) driving the expression of MRs using specific promoters. To date*, CREATES RECOMBINATION* (*CRE*) flanked by loxP sites has been the recombination system of choice for the excision of MR genes from expression cassettes (Lowe et al., 2016; Mookkan et al., 2017; Zhang et al., 2019; Hoerster et al., 2020; Masters et al., 2020). The transformation vectors include a region containing both the MRs and CRE/loxP, whose expression is driven by inducible promoters that can be triggered after the first steps of somatic embryogenesis induction to excise the MR cassette (Figure 2B). Examples of inducible promoters are those driving the drought-responsive *Rab17* gene (Lowe et al., 2016; Mookkan et al., 2017; Zhang et al., 2019) or the heat-inducible *Hsp17.7* gene (Masters et al., 2020). The selection of successful excision events may be facilitated by a fluorescent protein, which is only expressed after full excision of the CRE/loxP cassette.

A potential downside factor is that the quality of events may be affected because recombination is hardly 100% efficient and may leave part of the MR cassettes in the genome of the transformed events. Nevertheless, the frequency of complete recombination in transformed events ranges from 61 to 83% (Zhang et al., 2019). Another method avoids such drawbacks using specific promoters to drive MR expression. The rationale of this method is to maintain the expression of MRs in calli, embryos, and young leaves while avoiding its expression in meristems, roots, and reproductive organs. The use of a maize phospholipid transferase protein promoter (ZmPLTP) and an auxin-inducible promoter (ZmAXIG1) to drive *BBM* and *WUS2* expression, respectively, allows the recovery of healthy transformed plants without excision of the MRs (Lowe et al., 2018). The MR induction of somatic embryos seems to be genotype independent, as observed with more than 22 inbred lines from DuPont Pioneer (Lowe et al., 2018). Moreover, MR methods also have the advantage of inducing somatic embryogenesis directly from the scutellum epidermis, which skips the initial stage of callus formation and thus halves the time needed for *in vitro* culture prior to transferring the plants to a greenhouse (Figure 2A; Lowe et al., 2018).

Recently, an alternative method was proposed to avoid the integration of MRs in the genome of transformed events by infecting immature embryos with two *Agrobacterium* strains: one harbouring a construct with the selectable marker and target genes, and the other harbouring the MR construct (Hoerster et al., 2020). Upon infection, transient expression from the MR cassette induces somatic embryogenesis, whereas only embryos containing the selectable marker and target gene constructs integrated into the plant genome are recovered. The expression of *WUS2* is driven by the PLTP promoter incorporating viral enhancers that increase the induction of somatic embryogenesis and counterselect embryos in which the MR construct is eventually integrated because the high expression of *WUS2* inhibits regeneration (Hoerster et al., 2020). In addition to *WUS2* and *BBM*/*ODP2*, other MRs, such as *GROWTH-REGULATING FACTOR 5* (*GRF5*) and chimeric *GRF-INTERACTING FACTOR 1* (*GRF4-GIF1*), have been shown to increase the recovery of transgenic events (Debernardi et al., 2020; Kong et al., 2020).

MRs have been helpful for CRISPR/Cas9 genome editing, which usually demands the screening of many edited events (Zhang et al., 2019; Barone et al., 2020). In addition, genome editing based on less efficient systems, such as homologous recombination (HR), can benefit even further from a higher embryo recovery rate (Barone et al., 2020). MR transformation has also been efficient for CRISPR/Cas9 ribonucleoprotein (RNP) gene editing. In this case, IZE is co-bombarded with the RNPs of interest together with DNA constructs containing the MRs (Svitashev et al., 2016). This approach allows the recovery of foreign DNA- and marker-free GE plants. MR-based ternary vectors built by combining different backbones, helper plasmids, and recombination systems have been shown to be effective for maize CRISPR/Cas9 genome editing (Zhang et al., 2019). The commercially available “ready-to-use” vector is compatible with Golden Gate cloning for assembling the sgRNA expression cassette (Zhang et al., 2019).

### Haploid-Inducer Mediated Genome Editing System

CRISPR-Cas genome editing (CGE) in plants has evolved enormously in the past few years. The first efficient CGE process in plants was demonstrated in 2013 by three independent groups, which edited genes in rice, wheat, *Nicotiana benthamiana*, and *Arabidopsis thaliana* (Li et al., 2013; Nekrasov et al., 2013; Shan et al., 2013). As a result, CGE has become the most accessible, efficient, and versatile genome-editing tool for plants (Chen et al., 2019). In general, CGE uses a Cas9 endonuclease and a chimeric single guide RNA (sgRNA) that drives Cas9 to a target DNA sequence in the genome (Charpentier and Doudna, 2013). A range of CRISPR-Cas toolkits have been made available for major crops (Chen et al., 2019), to be used for various aims, including the simultaneous editing of multiple traits and precise allelic replacements (Svitashev et al., 2015; Shi et al., 2017; Chen et al., 2019; Kelliher et al., 2019; Wang et al., 2019; Ahmar et al., 2020; Liu et al., 2020; Van Tassel et al., 2020; Zhang et al., 2020a; Gao et al., 2020b). However, a frequent bottleneck of the technique is the delivery of CRISPR-Cas by standard *Agrobacterium* or biolistic methods because most crop varieties are recalcitrant to transformation.

**Figure 3:**
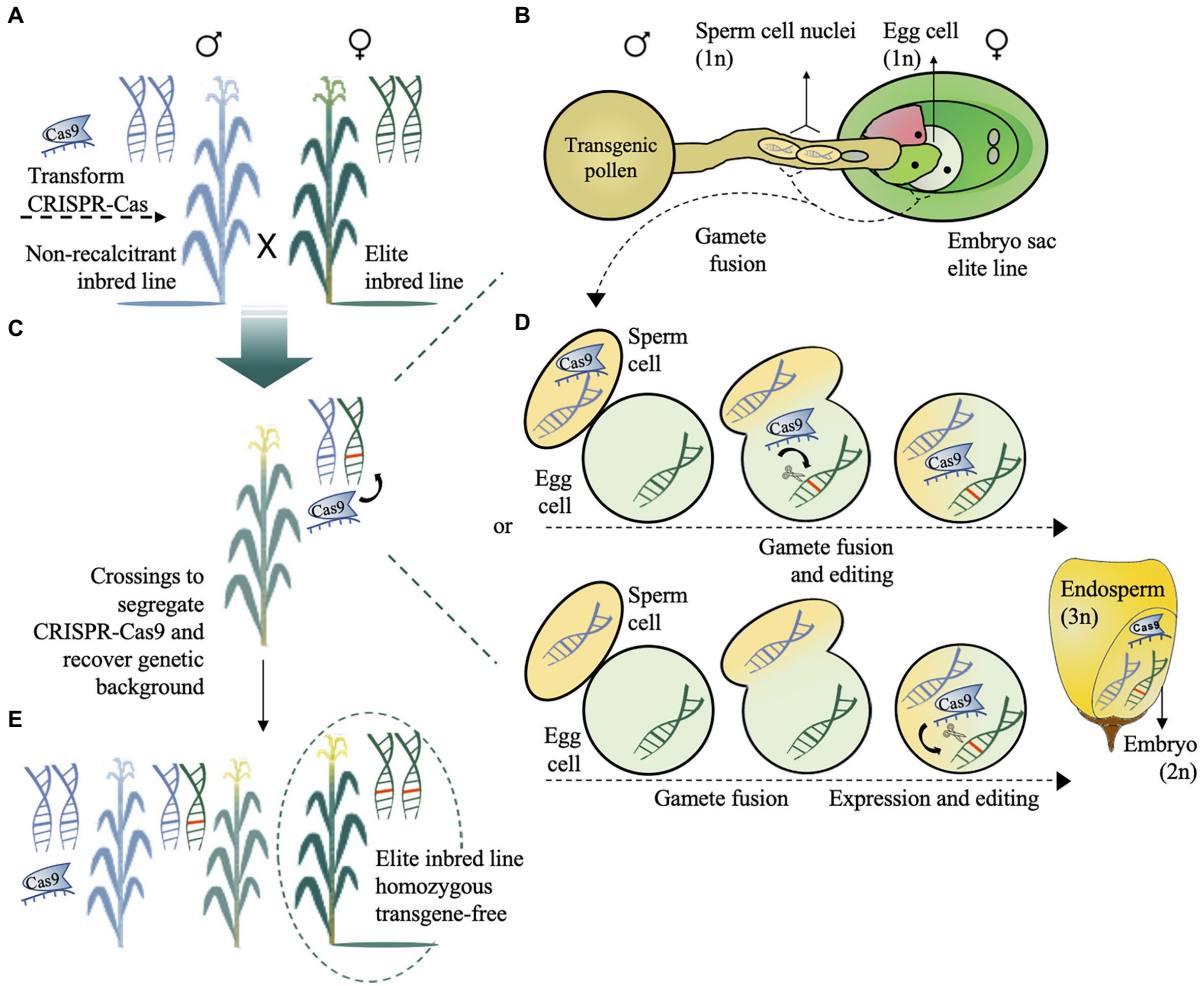
Schematic representation of the desired-target mutator (DTM) maize transformation. **(A)** The CRISPR-Cas cassette can be transformed into a nonrecalcitrant inbred line for *trans* editing in an elite recalcitrant inbred line. **(B)** Pollen carrying a CRISPR-Cas cassette designed to target gene(s) of interest was used to pollinate the elite maize line. **(C)** The target gene is directly edited *via* trans-acting CRISPR-Cas. **(D)** The delivery of RNP, which is expressed by the sperm cell, directly into the egg cell of the elite line (gametophytic expression) or expression of RNP in the zygote after gamete fusion (zygotic expression) generates a hybrid edited embryo. **(E)** After trans editing, subsequent crossings are needed to obtain CRISPR-Cas-free plants with the original receptor genetic background and homozygous to the desired mutation. The schematic illustration view in (D) was adapted from Jacquier et al. (2020).

**Figure 4:**
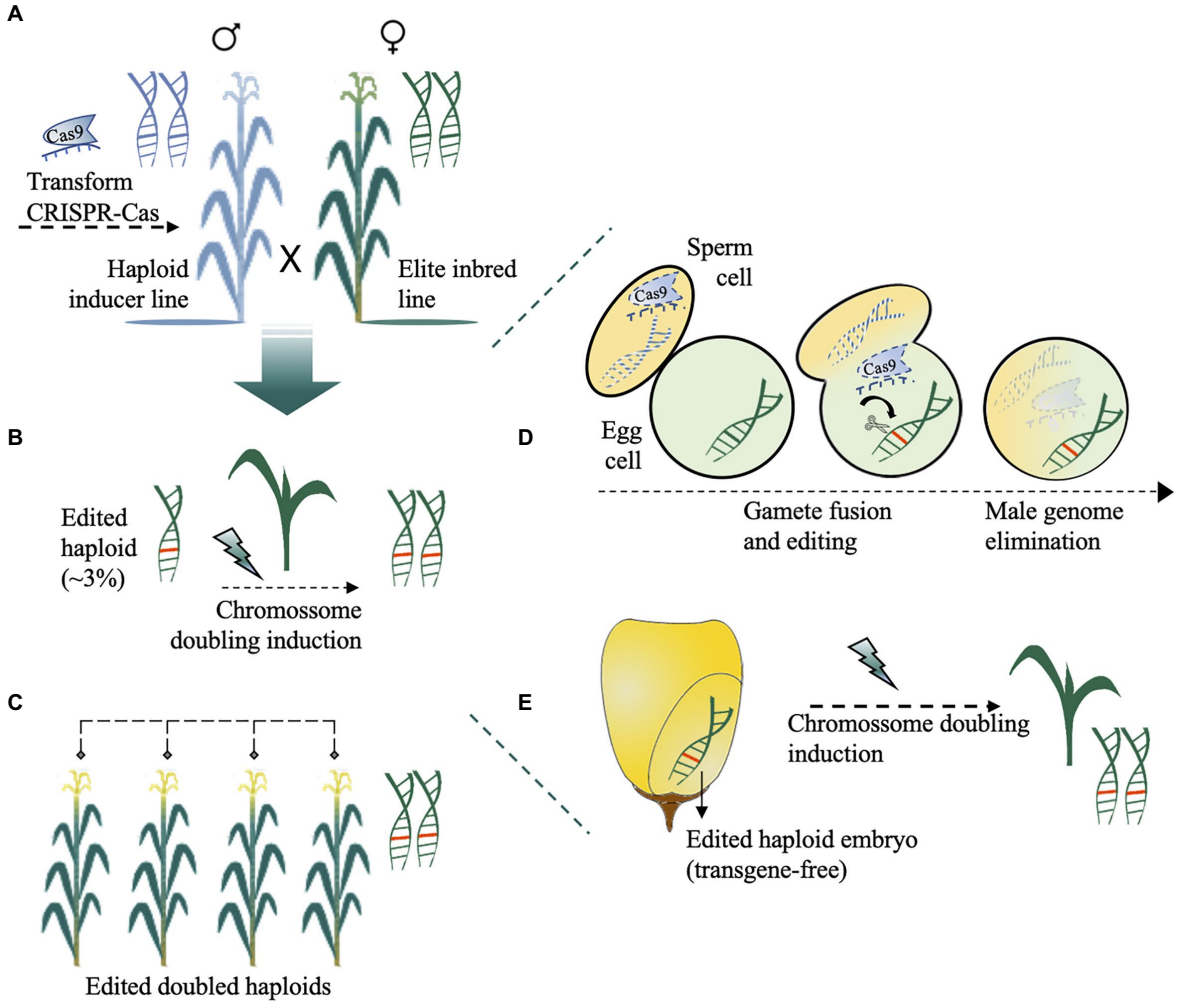
Haploid-inducer-mediated genome editing (IMGE) or simultaneous double haploid production and editing (HI-Edit) in maize. **(A)** The haploid inducer line harbouring a CRISPR-Cas cassette is used to pollinate the maternal elite line of any genotype. **(B)** The haploid progeny, which are typically sterile, were screened for CRISPR-Cas-induced mutations (~3%) and subsequently treated with a doubling agent to produce fertile doubled haploids. **(C)** Edited doubled haploid lines with improved agronomic traits are obtained after self-pollination. The zoomed view of *trans* genome editing and the maternal haploid formation processes occurring in B are shown in D-E. **(D)** After trans-acting CRISPR-Cas, and fertilization, the unstable paternal chromosome from haploid-inducer pollen is lost. **(E)** The formed embryo is nontransgenic (Cas-free) and has a doubled chromosome to recover the homozygous edited diploid elite plant.

Effective methods to deliver the CRISPR-Cas machinery in germplasm recalcitrant to gene editing by crossing have been proposed (Li et al., 2017; Kelliher et al., 2019; Wang et al., 2019). Direct genome-editing technologies, including desired-target mutator (DTM; Li et al., 2017; Qi et al., 2020) and HI-Edit (Kelliher et al., 2019), which is also known as IMGE (Wang et al., 2019), are based on the pollination of elite recipient inbred lines using the pollen of a stably transformed line harbouring a CRISPR-Cas construct (Figures 3–4). In DTM, the target gene is directly edited in a desirable allele *via* trans-acting CRISPR-Cas (Figures 3A–C). The *trans* editing can occur by (1) the delivery of Cas RNPs, which are expressed by the sperm cell, directly into the egg cell of the elite line (gametophytic expression) and (2) the expression of Cas and sgRNA in the zygote after gamete fusion (zygotic expression; Figure 3D). Both CRISPR-Cas systems depend on the promoter used to drive cassette expression (e.g.: pollen-specific promoter versus constitutive promoter, Jacquier et al., 2020). Thus, after subsequent crossing, CRISPR-Cas-free plants with the original receptor genetic background that are homozygous for the desired edited gene can be obtained (Figures 3C,E). Although backcrossings are needed to recover the original genotype, this method dramatically reduces the workload of introgression breeding programs that usually need marker-assisted backcrossing and more generations to minimize the linkage drag effect (Peng et al., 2014; Li et al., 2017).

In the HI-Edit/IMGE method, the paternal genome is a haploid inducer line harbouring a CRISPR-Cas cassette used to pollinate the maternal elite line (Figure 4A). In this case, the maternal genome is edited *in trans*, whereas the male genome is eliminated at the zygote phase. The haploid progeny, which is typically sterile, are then screened for CRISPR–Cas-induced mutations (Figure 4B) and subsequently treated with a colchicine mitotic inhibitor (Prasanna et al., 2012; Melchinger et al., 2016; Chaikam et al., 2019) or another less toxic doubling agent (Geiger and Gordillo, 2009; Häntzschel and Weber, 2010) to produce fertile doubled haploid and gene-edited nontransgenic plants (Kelliher et al., 2019; Wang et al., 2019; Figures 4C–E). However, improvements are needed to overcome the inherent problems related to haploid induction *per se* (Trentin et al., 2020; Jacquier et al., 2021). For example, CRISPR-Cas can be used before or in parallel to HI-Edit to: (1) increase the haploid induction rates by targeting genes related to high haploid induction (Kelliher et al., 2017; Zhong et al., 2019) or genes related to the inducer exclusion (Kelliher et al., 2019); (2) accelerate and accurately sort kernels/seedlings with haploid embryos from normal embryos by modifying visual traits such as anthocyanin (Chaikam et al., 2019) or by integrating visible transgenic markers into the inducers (Yu and Birchler, 2016; Xu et al., 2021; Yan et al., 2021) or even targeting genes involved in the oil content of seeds (Melchinger et al., 2013) and fixing recessive alleles of morphological traits in donors (Trentin et al., 2020). Care should be taken with desirable/undesirable agronomic traits during induction of haploid plants that would compromise the breeding programmes (Trentin et al., 2020). Desirable agronomic traits could be further targeted by gene editing, to take full advantage of HI-Edit/IMGE technology.

Overall, DTM and HI-Edit/IMGE technologies can help stack favourable genes in the genomes of elite lines. Precise genome modification overcomes the difficulties of traditional random uncontrolled mutagenesis and unpredictable insertions into the plant genome and thus has potential positive impacts on plant breeding (Schiemann et al., 2019). As a potential drawback, the CRISPR-Cas technology can generate undesirable off-targets but, although frequently predicted *in silico*, has been shown to occur rarely in plants (Young et al., 2019; Graham et al., 2020; Herbert et al., 2020; Gao et al., 2020a).

### Nanoparticle-Mediated Transformation

As discussed in previous sections, new technologies have pushed maize transformation (especially that aiming at genome editing) closer to a genotype-independent status in the past few years. These tools have enabled achieving levels of efficiency and scale that are practicable at academic and industrial settings.

Another promising technology employs nanoparticle-mediated (NP) delivery of macromolecules in a tissue culture-independent manner. The major advantages of NPs reside in their small sizes, diverse geometries, and versatile surface activation and binding capabilities (Cunningham et al., 2018). Although the exclusion limit of cell membranes is approximately 500nm, that of plant cell walls ranges from approximately 5 to 20nm (Cunningham et al., 2018) and is thus the major size-limiting barrier for introducing materials intracellularly. Carbon nanotubes (CNTs), a type of NP with a size of at most ~20nm in one dimension, can avoid the requirements for harsh entry methods that frequently result in cell damage. Indeed, the passive delivery of biomolecules into leaf cells of intact plants and the protection of polynucleotides from nuclease degradation have been observed with ≤12-nm single-walled carbon nanotubes (SWCNTs; Demirer et al., 2019). Furthermore, NPs can adsorb and carry a variety of cargo chemistries into cells, including DNA, RNA, proteins, RNPs, and small molecules (Cunningham et al., 2018), which makes NPs suitable for various approaches, which not only include genetic modification but also gene expression and pharmacological perturbations.

### Pollen Transformation

Tissue culture-independent methods for plant transformation in plants have been limited to a few species-tissue/cell type systems (for recent reviews, see Kausch et al., 2021a, 2021b and Anjanappa and Gruissem, 2021). One of these methods relies on delivering DNA constructs into mature pollen that is then used for direct pollination (Eapen, 2011). However, the methods remain controversial due to their low frequency or lack of reproducibility. In the case of maize, the aeration of pollen grains at 4°C in sucrose before sonication results in improved transformation (Yang et al., 2017). However, no successful transformation using this method has been published thus far.

Methods that exploit both the pollen biology and NP features are anticipated to result in significant improvements towards the realization of tissue culture-independent transformation of maize, particularly with respect to genome editing (Demirer et al., 2021), for which transgene integration is often unnecessary. In particular, subsequent advances are expected to take advantage of (1) the rapid and vigorous growth of maize pollen tubes both on stigma and *in vitro* (2) mounting knowledge of pollen and pollen tube gene expression and cell wall biochemistry (Zhou et al., 2017), which can inform further perturbation of their permeability, and (3) the versatile and customizable physicochemical properties and sizes of NPs, which would allow diverse cargo and passive cell entry.

## Development of Genetically Modified and Edited Varieties

### Biotechnology Pipeline for Trait Development

Over the last three decades, genetically modified (GM) maize varieties have successfully reached the market, which has brought traits such as herbicide resistance and insect resistance to farms. The first generation of varieties incorporated a single gene with only one mode of action against one insect order for insect resistance. The following generations were obtained by crossing herbicide and insect resistant events and different insect resistance events to achieve multiple modes of action against different insect orders. These so-called stacking varieties have reached impressive success with farmers, as demonstrated by a clear and complete phenotypic outcome (ISAAA database, 2021). The development of these first-generation traits is somewhat obvious because the science behind this development was described very early in the literature. However, the related process for quantitative traits such as abiotic stress tolerance, nutrient use efficiency, and yield is substantially more complex because these traits involve multiple genes that are subjected to strong environmental influence. To explore the impact of single genes on complex traits, companies have developed programmes ranging from gene to field biotechnology pipelines to evaluate gene effects at a large scale (Simmons et al., 2021).

**Figure 5:**
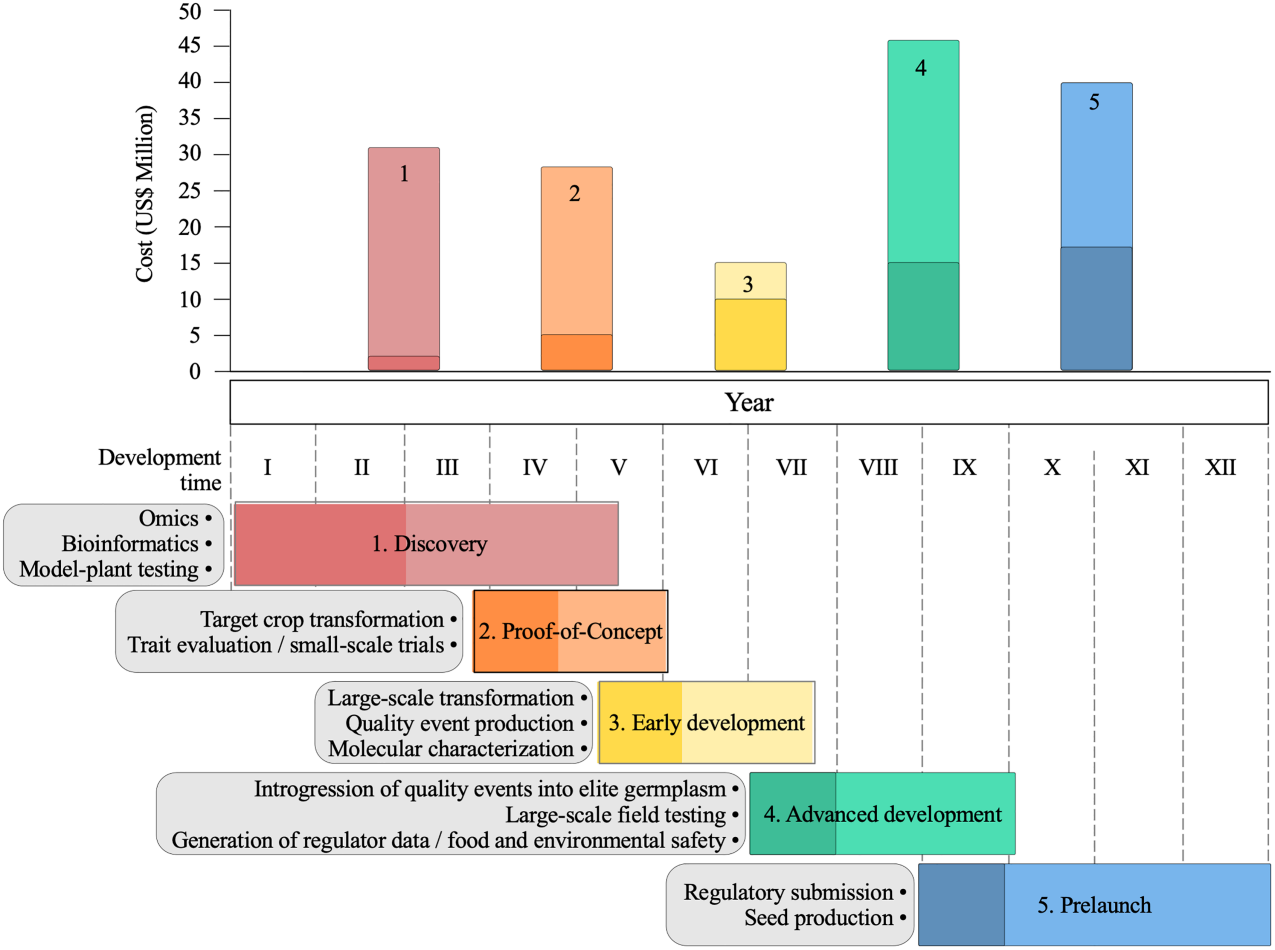
Agricultural biotechnology pipeline for trait development in maize. General overview of main activities and estimates of maximum (light colours) and minimum (dark colours) costs and development time of each pipeline phase: discovery, proof-of-concept, early development, advanced development and prelaunch. Estimates are based on Mcdougall (2011) and Mumm (2013).

A typical biotechnology pipeline (Mumm, 2013; Prado et al., 2014; Simmons et al., 2021) involves the following phases: discovery, proof of concept, early development, advanced development, prelaunch, and launch of commercial varieties (Figure 5). Some phases have activities that overlap temporarily, particularly when a positive early discovery lead is found, and the optimization activities begin before the end of the validation. The gene discovery phase is challenging, costly, and uncertain, particularly for traits such as drought and yield, which require well-defined phenotypic responses to drive the search for candidate genes (Nuccio et al., 2018; Simmons et al., 2021). High-throughput phenotypic screening of model plants, usually *A. thaliana* and *O. sativa*, is used to test hundreds of candidate genes. The proof-of-concept phase is characterized by the generation of events for each candidate gene and the initial phenotypic testing in both controlled environments and small-scale field trials (Simmons et al., 2021). At the end of this phase, maize events showing good agronomic performance, stable trait expression, and inheritance are elected as leads. In the early development phase, lead optimizations to improve the stability and enhance protein expression are usually required, and the leads are molecularly characterized and tested in large-scale field trials on multiple target locations and years (Simmons et al., 2021). The advanced development phase is characterized by the introgression of validated leads on commercial lines, and this process often involves the use of molecular markers to accelerate the breeding process and ensure trait conversion (Mumm, 2013). Regulatory data on gene product toxicity, allergenicity, compositional analysis, and environmental and human safety are also generated. In the prelaunch phase, the number of seeds from the new GM variety are increased to reach the market, quality control is implemented to secure trait identity and purity, a regulatory report is submitted, and the new GM trait hybrid is prepared for commercial launch. Depending on the trait and the resources available, completion of the pipeline takes, on average, 11–13years (Mcdougall, 2011; Mumm, 2013; Nuccio et al., 2018).

The average cost to develop a commercial maize GM trait is estimated at $50–136 million (Figure 5; Mcdougall, 2011; Mumm, 2013). Excluding the discovery phase, which can vary depending on the target trait, the advanced development and prelaunch phases are costly and time-consuming (Figure 5) because multiple field trials and regulatory studies are needed to ensure the safety and quality of the developed GM variety. However, genome editing that is free from transgene DNA sequences and is already regulated as non-GM in several countries would significantly contribute to decreasing the cost of regulatory studies and the overall costs of launching commercial varieties (Figure 5; Lowe et al., 2016; Lassoued et al., 2019).

### Regulatory Issues Associated With Maize GM Traits

Since the release of the first commercial insect-resistant GM maize 25years ago (Tabashnik and Carrière, 2017; Pellegrino et al., 2018), 148 GM maize events have been approved for commercial use worldwide (ISAAA database, 2021). The global area cultivated with GM maize occupied 61 million hectares in 2019, and this area included 33 million hectares in the USA, 15 million hectares in Brazil, 6 million hectares in Argentina, and 2 million hectares in South Africa (ISAAA Brief, 2019). However, although GM crops pose no additional risks to humans and the environment compared with conventional crops (National Academies of Sciences, Engineering, and Medicine, 2016), the public constraints of the technology, particularly in the European Union, remain high (Woźniak et al., 2021). The development of new plant breeding technologies (NPBTs), such as cisgenesis, intragenesis, and genome editing, can contribute to modifying public perception, particularly if they are properly communicated to society (Cardi, 2016; Harfouche et al., 2021).

The availability of plant genomes for major crops and their wild relatives will allow the identification of genes underlying the traits of interest and their precise modification or transfer into targeted varieties (Michael and VanBuren, 2015; Cardi, 2016). In this regard, cisgenic and intragenic mutations that are based on genetic alteration within a crop genome or the transfer of genes from sexually compatible species may illuminate an amenable regulatory path (Holme et al., 2013; Schaart et al., 2016). Although the current regulatory path regarding the biosafety of cisgenes/intragenes remains complex, some of the end products are indistinguishable from conventional plant breeding products. In Australia, Canada, India, and the USA, these products are reviewed on a case-by-case basis, whereas in the European Union, cisgenic and intragenic plants are regulated as regular GM organisms (Hull et al., 2021). Although no commercial cisgenic or intragenic maize has been launched to date, studies based on the genome-wide association and genome sequencing of maize lines will certainly lead to the identification of genes with the potential to improve cultivated genotypes using a cisgene/intragene approach (Hufford et al., 2021).

Genome editing by site-directed nuclease (SDN) technologies has the potential to be widely accessible to the scientific community for the generation of biotechnology crops (van de Wiel et al., 2017). SDN applications can be divided into SDN-1, SDN-2, and SDN-3 (Grohmann et al., 2019). SDN-1 and SDN-2 generate small-sized random (SDN-1) or template-directed (SDN-2) mutations at predefined genomic loci and have thus been considered mimics of those resulting from natural DNA variation (Eckerstorfer et al., 2021). Thus, a handful of countries consider crops modified by SDN-1 and SDN-2 as conventional plants (Anders et al., 2021). Argentina, Chile, the USA, Canada, Brazil, Colombia, and Paraguay have already approved normative resolutions on genome editing (Eckerstorfer et al., 2019), whereas the European Union relies on the legislation of GM organisms to restrict the cultivation of genome-edited crops (Turnbull et al., 2021).

### Currently Approved Commercial Maize Events and Future Expectations

The adoption of GM traits is considered the fastest innovation embraced by farmers around the world. From 1996 to 2018, the global economic gains from GM crop varieties reached US$ 225 billion, and 52% of these gains were in developing countries (Brookes and Barfoot, 2020). In 2019, transgenic plants were cultivated on 190.4 million hectares in 29 countries for consumption as food and feed, and this amount represents a 112-fold increase from 1.7 million hectares in 1996. Among the most adopted crops, soybean stands out, followed by maize, cotton, and canola. Of the 193.4 million global maize cultivation areas in 2019, 31% (60.9 million hectares) in 14 countries cultivated GM maize varieties (ISAAA Brief, 2019). Currently, maize is the crop with the most approved GM events, and 148 (36.2%) events in 35 countries mostly combine insect resistance and herbicide tolerance traits (ISAAA Brief, 2019). Other GM traits already commercially approved for maize are the restoration of fertility, male sterility, increased drought tolerance, production of phytase, modified amino acids and alpha amylase, enhanced photosynthesis, and increased ear biomass. These approved traits represent 39 single genes (Supplementary Table S1), and the majority of these genes are related to insect (18) and herbicide tolerance (11). The next generation of GM maize varieties potentially coming to the market comprises events with new insecticidal proteins such as Vpb4Da2, DvSnf7 RNA, and IPDO72Aa to control the population of insects already resistant to Bt (Schellenberger et al., 2016; Moar et al., 2017; Yin et al., 2020), varieties that exhibit improve grain yield by overexpressing the *zmm28* and *ZM-BG1H1* genes (Wu et al., 2019; Simmons et al., 2020) and varieties that exhibit tolerance to drought stress by overexpressing ARGOS8 (Shi et al., 2015).

The first and only commercial genome-edited maize variety was developed by Corteva to produce a high content of amylopectin (Gao et al., 2020a). Drought stress-tolerant genome-edited maize has been developed by precise modification of the promoter region to increase the expression of the *ARGOS8* gene (Shi et al., 2017). Other genome-edited maize varieties currently being developed include varieties with the traits of male sterility to facilitate hybrid development (Wan et al., 2019), tolerance to multiple stresses (Zhou et al., 2016), and dwarfism (Zhang et al., 2020b). From 2018 to 2020, several companies invested in genome editing in maize for achieving drought tolerance, resistance, and increased yield and stability, in addition to several traits investigated by government or academic institutions (USDA-APHIS, 2021).

## Concluding Remarks

In this review, we discuss the recent advances in genetic transformation and its application to introducing genetically modified and edited maize events to the market. Suitable maize transformation protocols using particle bombardment and *A. tumefaciens* are available and extensively applied for maize transformation. B104 is the most a suitable genotype, particularly due to its good agronomic performance, which directly allows the production of hybrids for field trial evaluation. However, transformation and plant regeneration remain limiting factors to the generation of elite commercial maize lines. This barrier should be transposed soon through the use of genotype-independent MRMT, which bypasses the callus phase and allows the rapid induction of somatic embryos from transformed scutellar cells. The advances in maize transformation technologies have also allowed the devising of strategies such as Hi-EDIT/IMGE to accelerate the genome editing-driven breeding of elite maize germplasms. Thus, platforms for advanced maize biotechnology and breeding by combining genomics and genome editing and the discovery of genes and alleles for complex traits will certainly allow the development of varieties that are better adapted to the biotic and abiotic stresses imposed by global climate changes.

The adoption of genetically modified maize varieties is already consolidated and has been shown to increase crop yields, reduce pesticide and insecticide use, and decrease the cost of crop production. However, even though GM crops are the fastest technology adopted by farmers, their acceptance by consumers remains low. The emergence of new plant breeding technologies, at least those that do not incorporate foreign DNA into the host cell, can change consumer perception and increase food security strategies. It is hoped that the new technologies discussed in this review will enable the release of more significant numbers of maize lines carrying desired traits to meet today’s agriculture challenges.

## Author Contributions

JECTY, VCHS, JHL, RAD, IRG, FRF, PAS, LRV, VB, PA contributed with literature review and writing of different sections that compose this work. JECTY contributed with several topics, compiled topics produced by the other contributors, revised, and combined it into the final writing work. JHL and VCHS prepared the Figures. PA helped design the structure of the review and revised and defined the final version. All authors read and approved the final manuscript.

## Funding

We are grateful to the Fundação de Amparo à Pesquisa do Estado de São Paulo (FAPESP) for supporting this research under the project “The Genomics for Climate Change Research Center (GCCRC),” grant no. 2016/23218-0. This study was funded in part by the Empresa Brasileira de Pesquisa Agropecuária (Embrapa) and the Universidade Estadual de Campinas (Unicamp). VCHS received a FAPESP postdoctoral fellowship (grant no. 2018/06442-9). JHL received a postdoctoral fellowship from Embrapa and Conselho Nacional de Desenvolvimento Científico e Tecnológico (CNPq; 381669/2019-0). LRV received a FAPESP postdoctoral fellowship (grant no. 2020/10677-1). VB received a FAPESP postdoctoral fellowship (grant no. 2020/09007-1). PAS is supported by Sempre Sementes S/A. IRG is a CNPq research fellow.

## Conflict of Interest

The authors declare that the research was conducted in the absence of any commercial or financial relationships that could be construed as a potential conflict of interest.

## Publisher’s Note

All claims expressed in this article are solely those of the authors and do not necessarily represent those of their affiliated organizations, or those of the publisher, the editors and the reviewers. Any product that may be evaluated in this article, or claim that may be made by its manufacturer, is not guaranteed or endorsed by the publisher.
